# Low-Loss Photonic Reservoir Computing with Multimode Photonic Integrated Circuits

**DOI:** 10.1038/s41598-018-21011-x

**Published:** 2018-02-08

**Authors:** Andrew Katumba, Jelle Heyvaert, Bendix Schneider, Sarah Uvin, Joni Dambre, Peter Bienstman

**Affiliations:** 1 Photonics Research Group, Department of Information Technology, Ghent University - imec, Ghent, Belgium; 2 IDLab, Department of Electronics and Information Systems, Ghent University - imec, Ghent, Belgium

## Abstract

We present a numerical study of a passive integrated photonics reservoir computing platform based on multimodal Y-junctions. We propose a novel design of this junction where the level of adiabaticity is carefully tailored to capture the radiation loss in higher-order modes, while at the same time providing additional mode mixing that increases the richness of the reservoir dynamics. With this design, we report an overall average combination efficiency of 61% compared to the standard 50% for the single-mode case. We demonstrate that with this design, much more power is able to reach the distant nodes of the reservoir, leading to increased scaling prospects. We use the example of a header recognition task to confirm that such a reservoir can be used for bit-level processing tasks. The design itself is CMOS-compatible and can be fabricated through the known standard fabrication procedures.

## Introduction

The surge in the volumes of data generated and consumed by devices has pushed the bounds on the scalability and speed of signal processing and computational systems. The consequence is a re-ignition of research into non-conventional information processing techniques that deviate from the well-established von-Neumann approach.

Analog computing has been proposed as a promising implementation in this regard in a number of alternative platforms. Analog computing, unlike conventional computing, relies on the information processing capabilities of certain physical systems. The processing usually hinges on the evolution of the internal state of the dynamical behavior of the physical system in response to an appropriately encoded input.

Analog information processing platforms are typically coupled with an adaptation and readout system to guide the evolution of the system state (or a subset of it) towards a solution whose accuracy metric is usually known beforehand. This is then followed by a readout that transforms the observed physical signal of interest into a form that is suitable for interpretation by digital computers. A prevalent way of implementing the adaptation of the computational system’s internal states is through machine learning.

Machine learning encompasses a set of techniques to teach computer systems how to perform complex tasks on previously unseen data, without explicitly programming them. Examples of tasks that are suitable for some form of machine learning are classification, regression or pattern recognition. The collection of machine learning techniques is indeed extensive and for every application the most appropriate technique has to be selected, depending on the application’s specific demands.

One important class of techniques are the so-called artificial neural networks (ANNs), that consist of a network of interconnected computational units, dubbed neurons. The layout and operation of the ANN is inspired by the structure and information processing mechanism of the human brain. Recurrent neural networks (RNNs), a subtype of neural networks, introduce memory into the network by creating directed interconnection cycles between neurons in order to tackle tasks with temporal extent.

**Figure 1:**
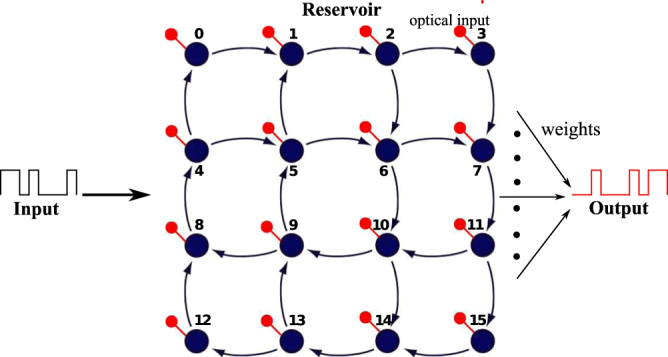
Schematic of a photonic reservoir computing setup for handling tasks involving digital optical signals. The input is a non-return-to-zero on-off-keying (NRZ-OOK) digital optical signal, the reservoir is composed of 16 nodes arranged in a swirl topology and the output is read from each node through a photodector. From here on, nodes (blue filled circles) will be referenced by their corresponding labels in the illustration. In the swirl architecture as is used here, the nodes are the locations at which states are appropriately combined and split and serve as input and detection points (red circles).

Reservoir computing (RC)^1–3^ was proposed as a methodology to ease the training of these recurrent networks, which was typically rather challenging. More recently, however, it has gained popularity as a computational paradigm to solve a variety of complex problems. It has been shown that RC performs very well on e.g. speech recognition and time series prediction. Reservoir computing initially emerged as a software-only technique and merely presented another algorithmic way of processing temporal data on digital computers. However, it has evolved into much more over the past decade. The RC system consists of three basic parts: the input layer which couples the input signal into a non-linear dynamical system, the “reservoir” (i.e. the recurrent neural network, which is kept untrained) and finally the output layer that typically linearly combines the states of the reservoir to provide the time-dependent output signal. An illustration of the reservoir computing system as is applied in this work is given in Fig. 1. To use the reservoir to solve a particular task, a machine learning algorithm is used to train a set of weights (the readout) using a set of known labeled example data, such that a linear combination of the optical signals recorded at each node approximates a desired output as closely as possible. These weights are then used to generate the output signal for any unseen subsequently injected input signal sequences. RC systems are fast to train and quickly converge to a global optimum. They have shown state-of-the-art performance on a range of complex tasks on time-dependent data (such as speech recognition, nonlinear channel equalisation, robot control, time series prediction, financial forecasting, handwriting recognition, etc.).

A key discovery was that the reservoir computing platform provides a natural framework for implementing hardware-based learning systems for which there may be only a limited ability to granularly influence the internal state of the dynamical system (reservoir). Examples of RC implementations in mechanical systems, memristive systems, atomic switch networks, boolean logic elements and photonic systems can be found in^4–8^.

To date, experimental demonstrations of photonic reservoirs routinely achieve state-of-the-art performance on various information processing tasks. Implementations based on a single nonlinear node with a delayed feedback architecture have proven that photonic RC is competitive for analog information processing^9–17^. Moreover, integrated photonic reservoirs can push computation speeds even higher for digital information processing. The performance of integrated photonic reservoirs has been studied numerically for networks of ring resonators^18–22^, networks of SOAs^7^, and experimentally with networks of delay lines and splitters^23^. Integrated photonic reservoirs are particularly compelling, especially when implemented in the CMOS platform as they can take advantage of its associated benefits for technology reuse and mass production.

However, while possessing numerous benefits, passive integrated photonic reservoirs are plagued by a number of issues, key among which is loss accumulation. Silicon photonics reservoirs are composed of nodes that are interconnected together in a planar topology such as that in Fig. 1. Since the interconnections between nodes are made up of spirals of a few centimeters, the material loss – ≈2 dB/cm for single-mode 220 nm Si waveguides – is important. An equally significant source of signal loss is the loss at combiner points. Indeed, based on supermode theory, combining single-mode waveguides in a Y-junction only has 100% transmission if the two inputs are exactly in phase. For anti-phase inputs, the transmission is 0%. Therefore, averaged over all possible phase differences, there is only a 50% transmission for each combiner traversed. An alternative way of expressing the same fact is by saying that if we only excite a single input of the combiner, we will have 50% transmission (Later on we will use this single-side excitation as a quicker way to model the average transmission for different phase differences). The 50% loss can quickly reach substantial values for large reservoirs with a lot of combiners. Using directional couplers instead of Y-junctions would in theory solve these issues, but they suffer from stringent fabrication tolerance requirements and narrow bandwidth. Both this combination loss and the propagation loss constrain the size of the reservoirs and hence limit the complexity of tasks they can tackle.

In this work we present an efficient passive photonic reservoir computing system that lowers the combination loss. In so doing, the system allows for upscaling the number of nodes in the design as loss build-up can be limited. Even a relatively modest improvement in trans-nodal transmission will yield a substantial overall gain, since splitting and combining of signals occurs a multitude of times before the signal is read out. This improvement hinges on using broader waveguides that hence support multiple modes.

One way to take advantage of multiple modes in reservoirs is by considering them as separate channels of computation and reading them out separately through a demultiplexing structure such as a cascade of asymmetrical directional couplers^24^. The total number of modes supported by the network represents the factor of increase of the number of observables in the system and is an indicator for the performance improvement. A multimode reservoir also directly implies richer dynamics due to multiple mixing avenues that could happen between the modes. However, this approach is not the focus of the current work but will be investigated in a follow-up study.

A second way to take advantage of the multimodal character is the one that we pursue here, where we will focus on loss reduction. A critical component in this design is a novel multimode Y-junction structure that is used at the combiner/splitter points. The junction uses a taper section that is deliberately designed to be not perfectly adiabatic, ultimately resulting in energy efficiency benefits. Note that multimodal photonics is something which is typically not considered for other applications, because it leads to several complications (modal dispersion, more complex design, difficulty to selectively excite and maintain a select number of modes throughout the whole circuit, …). However, in the context of the RC computing paradigm, none of these are of any consequence.

The key advantage from using the multimode Y-junction comes from the fact that a portion of the light that was previously scattered into radiation modes of the single-mode structure can now be captured into the higher-order guided modes. Indeed, in a case where e.g. light is only sent into one of the two input arms of a single-mode Y-combiner, when entering the output waveguide 50% of the light radiates away, as mentioned previously. However, if the output waveguide of the combiner supports multiple modes, this transmission increases to 100% for a perfectly adiabatic taper. Still, since part of this combined light is now carried by a higher-order mode as opposed to by the fundamental mode, it is still a fact that this higher-order light will radiate away at the next combiner. This is where the next part of the design comes into play: by deliberately designing the Y-junction to be non-adiabatic to a certain extent, we can hope to get a degree of conversion from these higher-order modes back to the lower-order modes which can propagate unhindered. Still, this beneficial process competes with the harmful reverse process of conversion from lower-order to higher-order modes. Therefore, it is not a-priori obvious that there will be design that offers a bigger than 50% transmission averaged over different modal compositions of input excitation. The main contribution of this paper is to present exactly such a design.

## Results

### Design of a multimode Y-junction with low combiner loss

#### Waveguide cutoff conditions

To effectively design multimode waveguides and components, we must first determine the cutoff conditions for different modes. This has to be done numerically, as the well-established closed form 1D solution cannot easily be extended to practical 2D cases. Lumerical^©^ Mode Solutions, a commercial mode solver package, was used to determine the modes of the waveguides. The fully vectorial FFM solver in Fimmwave^©^, which is based on the Film Mode Matching Method, was used to check the results for consistency.

**Figure 2:**
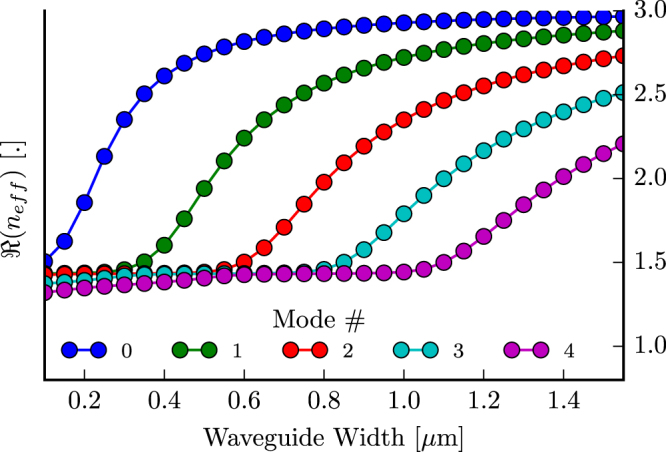
Dispersion diagram for 220 nm SOI waveguide for the TE polarization and a center wavelength of 1300 nm. *n*_*eff*_ is the complex effective index of the mode.

For this work we assume oxide-clad 220 nm silicon at 1300 nm on the SOI material platform. From here on, we will only focus on the TE-like modes but a similar argument applies to TM-like modes (we also confirmed that there was negligible interaction between the two mode groups). From the mode simulations we obtained the cut-off points for each mode and can therefore choose what width to use in order for the waveguide to support a given number of modes (Fig. 2).

#### Relevant geometrical parameters of Y-junction

**Figure 3:**
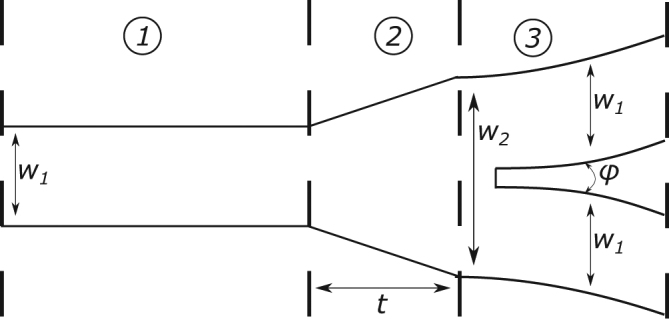
Sketch of the Y-junction indicating the sections critical to its performance.

The Y-junction design for the simulations was composed of three sections as seen in Fig. 3. The input waveguide (section 1) of width *w*_1_ is followed by a linear taper of length *t* (section 2) leading into a wider waveguide with width *w*_2_. The third section starts off right after the taper and terminates into a split into the two output waveguides. The output waveguides (also referred to as arms) are all the same width *w*_1_ as the input waveguide of section 1. *ϕ* is the angle between the two output waveguides and is determined by the bend radius of the output waveguides. The smaller the bend radius, the larger the value of *ϕ* and vice versa.

We shall refer to the splitter configuration as the case when the input is on the left side of Fig. 3 in section 1. For the combiner configuration, the junction is operated in the reverse sense.

In contrast to the single objective criterion of maximizing adiabaticity for the single-mode Y-junction, designing a low loss multi-mode Y-junction combiner is a more intricate procedure, as the interactions and evolution of the modes through the junction drive the choice of the best geometrical parameters. Since sweeping all the geometric parameters would be very time-consuming, the simulations proceeded as follows. For all designs considered, *w*_2_ = 2*w*_1_, and the bend radius for the two arms is set to 5 μm. Subsequently, the taper length *t* was fixed and the width of the input and output waveguides *w*_1_ was scanned in the range 600 nm–1200 nm. Then, the taper length *t* was optimized to achieve maximum transmission for the Y-junction with the selected width.

#### Waveguide Width Optimisation

A first relevant parameter is the input and output waveguide width *w*_1_ for the Y-junction, for which we will compare four values, inspired by different regimes in the dispersion diagram of Fig. 2. At 600 nm width, the first two modes (i.e. fundamental and first-order mode) are strongly guided. A third mode exists, but is guided less strongly. At a width of 800 nm, the first three modes are well-guided, but now a fourth mode (i.e. third-order mode with effective index of 1.46 with a cladding index of 1.4469) is weakly guided. The situation for a 1000 nm wide waveguide has evolved further towards four well-guided modes and a fifth (i.e. the fourth-order mode) that is barely guided with an effective index 1.4495. The last design, with a width of 1200 nm, guides this fourth-order mode better and does not support the fifth-order mode yet.

The transmission was then simulated for these Y-junction designs through propagation simulations. For this purpose Lumerical^©^ FDTD solutions, a commercial FDTD software package, was used.

If we consider the transmission results from the simulations for the combiner configuration with input to the upper arm only as presented in Table 1, the gains of moving to wider waveguides are evident. Compared to the single-mode fully adiabatic combiner, we already have a small improvement in the transmission from 50% to at least 53.18% occurring in the 1000 nm–1200 nm waveguide width region.

**Table 1:** Transmission for Y-junction combiners of different input and output waveguide widths *w*_1_ when the input is a particular modal excitation.

width *w*_1_ (nm)	Combiner transmission (input to one arm only)					
	Fundamental	1^*st*^ order	2^*nd*^ order	3^*rd*^ order	4^*th*^ order	average transmission
600	89.14%	28.41%	0.29%	—	—	39.28%
800	76.84%	70.55%	17.36%	0.45%	—	41.30%
1000	89.99%	55.25%	55.02%	12.47%	—	53.18%
1200	93.47%	75.95%	17.49%	41.27%	3.97%	46.43%

The results here correspond to the case of excitation to the upper arm only. Missing values indicate that that particular mode is not guided for that waveguide width.

Each column in the table represents transmission values for excitation with a given input mode to a single arm only. The final column is an average transmission over all the previous columns for guided modes. Note that in a real reservoir, the modal composition at the input will take many different forms, and therefore this average transmission is used as a straightforward way to derive an approximate but relevant single figure-of-merit for the device, without having to deal with fully coherent multimodal simulations of an entire RC network. From the results, it is evident that for wider waveguides and junctions, the losses are substantially smaller than for smaller waveguides and junctions by virtue of the power ending up in higher order guided modes rather than being radiated away. As mentioned earlier, for the waveguide widths considered, the highest transmission is obtained in the 1000 nm–1200 nm range. One can argue that maximum transmission occurs in this region because it constitutes the widths at which power conversion from higher to lower order modes is most efficient for the given taper length *t* = 0.1 μm (*t* is chosen adhoc for this section). However, to reach the maximum of transmission for both the waveguide widths *w*_1_ and taper lengths *t*, we need to probe the influence of the taper length *t* on the transmission. This will be the focus of the *Taper Optimization* section below.

#### Modal power distribution

Before optimizing the taper length *t*, we first make a detour to check the output modal power composition of the multimode Y-junction with respect to input excitation of a particular mode. We focus on the case of a 1 μm wide Y-junction with a 0.1 μm taper. Table 2 represents the transmission from the upper arm into the input in the combiner configuration, while Table 3 shows the transmission from the input into upper arm in the splitter configuration.

**Table 2:** Modal decomposition of Y-junction combiner with 1 μm wide waveguides and 0.1 μm long taper for single excitation from upper arm.

Input source	Transmission from upper arm to input (combiner)				
	fundamental	1^*st*^ order	2^*nd*^ order	3^*rd*^ order	4^*th*^ order
fundamental	34.94%	41.94%	4.67%	5.01%	3.44%
1^*st*^ order	0.46%	6.38%	16.12%	20.72%	11.56%
2^*nd*^ order	5.07%	1.30%	26.43%	17.70%	4.52%
3^*rd*^ order	6.61%	0.05%	0.39%	2.90%	2.51%

**Table 3:** Modal decomposition of Y-junction splitter with 1 μm wide waveguides and a 0.1 μm long taper.

Input source	Transmission from input to upper arm (splitter)				
	fundamental	1^*st*^ order	2^*nd*^ order	3^*rd*^ order	4^*th*^ order
fundamental	34.95%	0.56%	5.05%	6.52%	0.36%
1^*st*^ order	41.90%	6.52%	1.36%	0.05%	0.01%
2^*nd*^ order	4.67%	16.10%	26.72%	0.39%	0.01%
3^*rd*^ order	5.06%	20.77%	17.88%	2.94%	0.48%

First, we observe that indeed several modes participate in the guiding of power through the junction, and that the distribution of power over the different modes is very different for different input configurations. We can also see some modal conversion taking place from input in higher-order modes to output in lower-order, better guided modes.

Second, the results allow us to verify the reciprocity of the device (to within simulation tolerances). For example, the transmission from the fundamental mode to the first-order mode in the combiner configuration in Table 2 is 41.94%, whereas the transmission from the first-order mode to the fundamental mode in the splitter configuration is 41.90% in Table 3.

#### Taper Length Optimisation

In the *Waveguide Width Selection* section above, we demonstrated that by going multi-mode we can gain a small improvement in the transmission of the Y-junction combiner. However, to continue to boost the transmission of the multimode Y-junction, and especially when averaged over many possible input modal excitations, we need to further tailor the adiabaticity of the evolution of the input modeset by making appropriate changes to the geometry. We chose to do this by altering the taper making up section 2 of the design. The simulations for the choice of the waveguide width were done with a taper length *t* of 0.1 μm, we now select the *w*_1_ = 1.0 μm design, which we found to be in the region of highest transmission in the first phase of our simulations, and track the transmission for taper lengths *t* = 0.1 μm, 1 μm, 2 μm and 2.5 μm.

**Figure 4:**
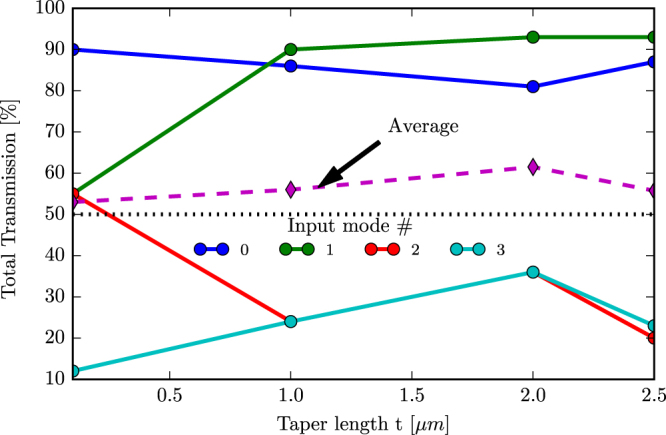
Total transmission (summed over all output modes) in the Y-junction combiner for different taper lengths. Results are shown for input to the upper arm of the junction consisting of the fundamental (0), 1^st^, 2^nd^ and 3^rd^ order modes as well as the average transmission across all input modes. The baseline transmission of 50%, for the adiabatic single-mode Y-junction, is also indicated.

The resulting transmission values for the multimode Y-junction combiner are plotted in Fig. 4 against taper length *t* for the case of excitation in the upper arm only. As input excitations, we use the fundamental, 1^st^, 2^nd^ and 3^rd^ order mode. Each solid line corresponds to the denoted input mode and at the output a sum of the transmission across all output modes is plotted as the total transmission. The average transmission across all input modes per taper length *t* is also plotted and is the same figure-of-merit as used in the last column of Table 1. As anticipated, the optimization has resulted in higher transmission values as can be seen by comparing with the results of the *t* = 0.1 μm case (which was used in a previous section to determine the best waveguide width for the multimode Y-junction) to the longer taper lengths.

As we alluded to earlier, there are two conflicting trends at work which result in an optimum average transmission occurring at taper lengths around 2.0 μm. On one hand, increasing the taper length increases the adiabaticity which decreases the scattering losses for the guided supermodes and increases the radiation loss of the unguided supermodes. (Note that also for this multimode junction, in the limit of a perfectly adiabatic taper we expect 50% average transmission: 100% transmission for modes 0 and 1, and 0% transmission for modes 2 and 3, which will couple to unguided supermodes). On the other hand, having a certain degree of non-adiabaticity is beneficial to convert some of the higher-order modes into better guided lower-order modes, as shown earlier. This is the mechanism that allows us to get above a 50% average transmission. When looking at the transmission of e.g. modes 2 and 3 as a function of taper length, there is no clear trend, indicating a complicated modal mixing and conversion taking place.

At this point, although we could further optimize the design, simulation results already show an average transmission of 61% for the combiner, which is much better than the standard 50% average loss in single-mode junctions.

As this is the design we will be using in the reservoir, we carry out further simulations to check its performance when operated in the splitter configuration and obtain 42% average transmission per output arm. While the loss in the splitter is higher than the 50% of the fully adiabatic single mode case, we will confirm later that the improvement in the transmission of the combiner will yield net gains in the power efficiency of the reservoir.

#### Wavelength dependence

**Figure 5:**
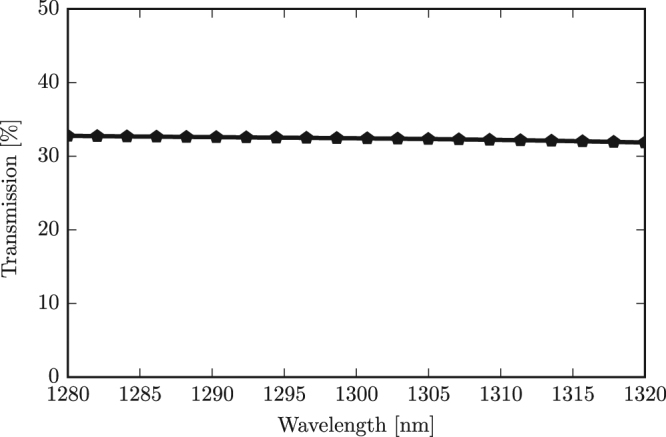
Influence of wavelength on the transmission of the multimode Y-junction with taper length 2 μm and waveguide width 1 μm for the fundamental mode.

We additionally evaluated the impact the wavelength of operation could have on the performance of a passive reservoir system that uses this multimode Y-junction design. Figure 5 tracks the transmission to the fundamental mode of the output Y-junction combiner from input excitation with the fundamental mode for various wavelengths, in a 40 nm bandwidth around the target wavelength of 1300 nm. Results are given here for the Y-junction of width *w*_1_ set to 1 μm and taper length *t* set to 2 μm. It can be seen that the curve is essentially flat and we can therefore conclude that variations in the wavelength will not impact the performance of the reservoir. Similarly flat curves were obtained for other input-output mode pairs.

### Performance of the improved Y-junctions in photonic reservoirs

We focused our numerical simulations on a model of a 16-node passive integrated photonics reservoir with the nodes arranged in a swirl topology^18^ (see Fig. 1). This architecture adheres to the planarity constraints of the CMOS Silicon Photonics platform while simultaneously allowing for sufficient mixing of the input signals.

As mentioned, in our design of the multimode Y-junction, we used as a figure-of-merit the average transmission across all considered excitations with different modes. Therefore, we need to setup our simulations in a way that matches with this scenario. Specifically, at all points where a combiner is needed we use 61% transmission, and similarly for all splitting locations we use splitters with 42% efficiency. Additionally, as we are taking average powers across all modes, our reservoir simulations will not be coherent (as is the case in most of our previous works) but will rather calculate the time evolution of the intensity of the input signals.

The reservoir was tasked to solve the 3 bit header recognition task. The specific details of the task encoding have been discussed in our previous work^18^.

#### Error rates for different data rates

We evaluated and compared the error rates of the reservoir on the 3 bit header recognition task for multiple data rates for single-mode and multi-mode reservoirs. The input data stream is an NRZ OOK modulated signal with an oversampling factor of 24 and the maximum considered data rate is 32 Gbps.

An total input power of 100 mW was used and for each data rate errors were obtained for 10 different random initialisations of the input bit stream and input weights.

**Figure 6:**
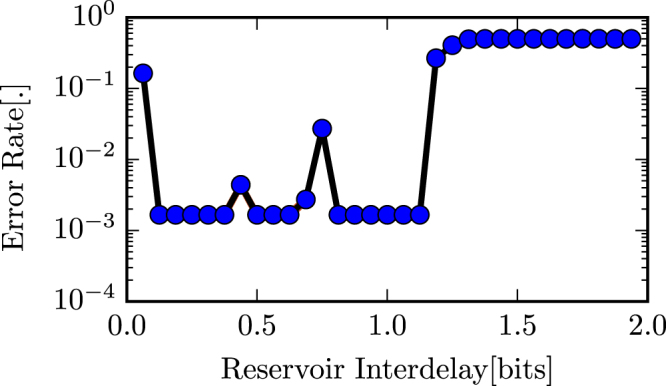
Error rate vs reservoir interdelay for the single-mode reservoir for the 3 bit header recognition task.

**Figure 7:**
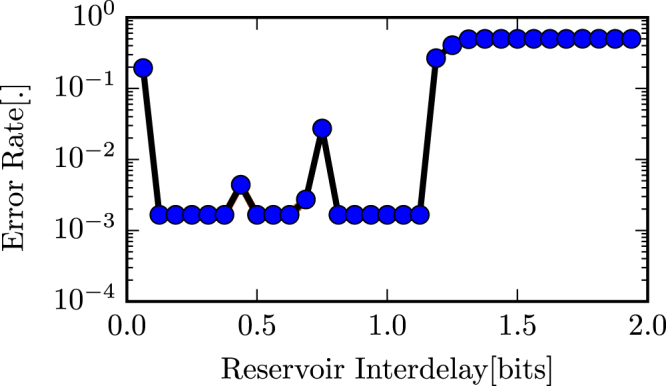
Error rate vs reservoir interdelay for the multimode reservoir for the 3 bit header recognition task.

Figures 6 and 7 show the results from the error rate vs reservoir inter-delay (a normalization of the data rate to the interconnection delay between 2 adjacent nodes) experiments for the single-mode and the multimode reservoir cases respectively, for the case of input to node 0. For the most part there is no significant difference in performance when going from single to multi-mode reservoirs, which means there is no performance hit associated to moving to multimodal Y-junctions. In both cases we have regions of performance below the Soft Decision Forward Error Correction (SD-FEC) limit (corresponding to a BER of 2 × 10^−2^) which means we are able to reach error-free performance by applying FEC codes. To see the real benefit of this work, however, we need to look at energy efficiency.

#### Energy efficiency considerations

**Figure 8:**
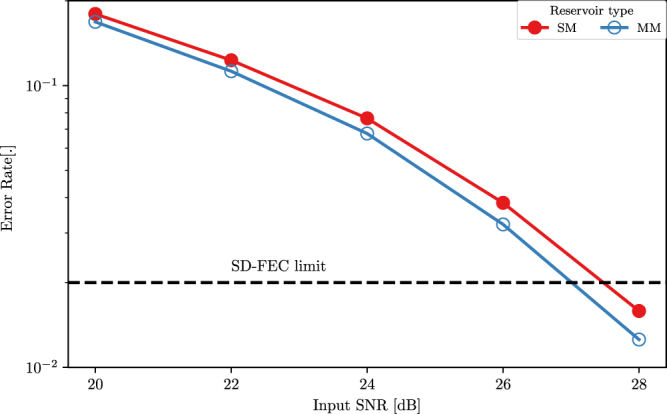
Error rates for a 16 node single-mode and a 16 node multi-mode reservoir on the 3 bit header recognition task for different values of the input SNR. The Soft Decision Forward Error Correction (SD-FEC) limit corresponding to an error rate of 2 × 10^−2^ is indicated.

The key goal of this work is to demonstrate that we can achieve energy efficiency benefits by replacing a single-mode reservoir with a multi-mode version. First, if we track the error rate for the single-mode and multi-mode reservoirs on the 3 bit header recognition task against the input signal to noise ratio as plotted in Fig. 8, we observe that errors for the multi-mode reservoir are always lower than those of the single-mode case. The trend also tends to show higher divergence for higher signal to noise ratios. While at the level of the SD-FEC limit the difference seems small, it should be noted that this diverging trend will persist as we characterize for even lower BERs.

**Figure 9:**
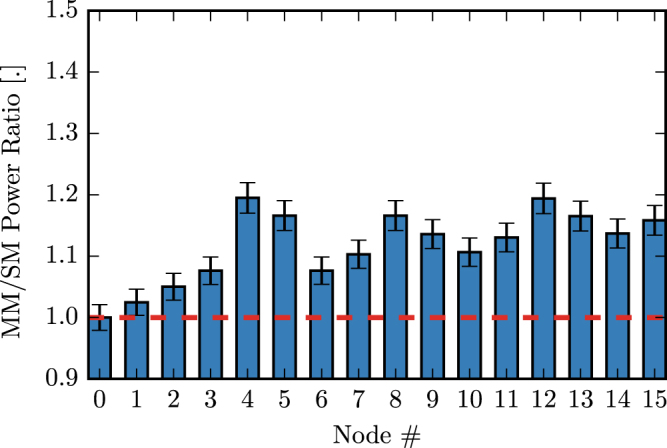
Comparison of single-mode and multimode 16 node reservoir average power per node for input to node 0.

Next, we compare the overall loss of a single-mode reservoir with that of a multimode reservoir. Figure 9 indicates the per-node power ratios for the 16 node passive reservoir for the multi-mode versus single-mode case for the case of input to node 0. This power ratio is obtained by dividing the average power of the states at each node in the multimode reservoir by the power of the same node in the single-mode reservoir and is shown for the 3 bit header recognition as we considered for the performance evaluation. Because node 0 is used as the input in both cases, its power did not change and consequently its power ratio is equal to one. All other nodes have a power ratio above the horizontal red line that indicates where the ratio is equal to one. This means that more power is measured at all those nodes in the multimode reservoir. We observe that we can get up to 20% more power in nodes such as 4, 8 and 12 (they also happen to be the furthest from the input node in terms of optical path length).

**Figure 10:**
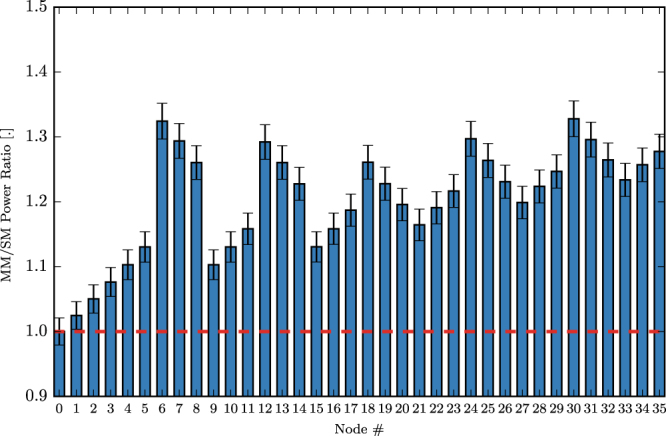
Comparison of single-mode and multimode 36 node reservoir average power per node for input to node 0.

We carried out one more experiment to further investigate how the improvement in the component energy efficiency affects the power distribution in the reservoir as we move to larger reservoirs. We re-simulated the 3 bit header recognition task but this time with a 6 × 6 (36 node) swirl reservoir. We observe, as shown in Fig. 10, that we now have nodes that have now more than 30% improvement in their power level (see e.g. nodes 6, 12, …). One can expect for the gains to increase even further as we move to larger networks. The additional power boost obtained for multimode reservoirs could be the difference between being below or above the noise floor and therefore could have significant impact on the performance and scalability of a real-world photonic reservoir computing setup.

## Discussion

Numerical simulations have shown that we can improve the overall energy efficiency of silicon photonics integrated circuit reservoirs by replacing the typical single-mode components and waveguides with multimode versions. We used FDTD electromagnetic simulations to show that we can design a low-loss multimode Y-junction with widths ranging from 500 nm onwards but with a preference for 1000 nm to keep the circuit size down while maintaining the advantageous aspects of mode evolution at such a junction. We found that the best designs can be implemented with taper lengths around 2.0 μm at an optimum level of adiabaticity. We showed that the best design can yield a combiner with an average transmission of 61% as opposed to the 50% in the best case single-mode junction combiner. The same device operating as a splitter gives 42% transmission, less than the 50% of the single-mode case, however when the device is used in a reservoir, the improvements in the combiner transmission make up for this reduction in the splitter performance. Further parameter optimization can be used to get even better improvements on the loss.

We have demonstrated that constructing a reservoir based on the average transmission characteristics for our optimal Y-junction combiner/splitter design yields the same task performance but gains in energy efficiency, when computing with intensities of the input signal. For single-node input to the reservoir, energy at some nodes could be enhanced up to as much as 30% in the case of a 36 node reservoir. This highlights how the small improvements in a single component design yield benefits that scale with the size of the reservoir. Such an improvement paves the way for building reservoirs with a larger number of nodes than is currently possible and that can therefore solve more complex tasks.

In future work we will explore the operation of the coherent multimode reservoir and design a photonics chip that implements the multimode reservoir computing paradigm to take advantage of the improved energy efficiency.

## Methods

### Numerical simulations

For calculating the eigenmodes of the waveguides, Lumerical^©^ Mode Solutions, a commercial mode solver package, was used. The Film Mode Matching Method-based solver in Fimmwave^©^ was used to double-check the results. For the Lumerical^©^ varFDTD method selected for the propagation simulations and optimisation of the parameters of the Y-junctions, an auto non-uniform meshing strategy was chosen at level 5 accuracy and a conformal variant 1 mesh refinement was applied. The center wavelength was set to 1300 nm and a silicon-oxide cladding of refractive index 1.4469 surrounding the 220 nm high silicon waveguide structure with material parameters according to Palik’s model in the Lumerical material database was used. The geometries of the structures were designed with Luceda Ipkiss^©^ Photonic Design Environment, exported into the GDSII format and then imported into Lumerical^©^. The varFDTD method was selected as it enables fast simulations (2D FDTD simulation speeds) with accuracy accuracy approaching that of 3D-FDTD simulations for devices with omni-directional in-plane propagation such as the MMI in this work.

### Circuit simulations and task setup

We use custom time-domain circuit simulation scripts based on the Caphe software^25^ and scikit-learn library^26^ for the machine learning. Unless stated otherwise, a 4 × 4 (16 node) reservoir architecture was used to generate the states. This number of nodes was chosen as it is a design that is both cost-effective to produce with multi-project wafer runs, but also has a good performance on a number of tasks. In all cases, the length of the interconnections between the reservoir translates to a propagation time of 62.5 ps, matching the current generation of experimentally available chips^18^.

Once the states were obtained and transformed with an appropriate photodetector model taking into account various noise contributions and bandwidth limitations, the readout was trained using the scikit-learn library^26^. For the training, 10,000 randomly chosen bits were fed into the reservoir and the resulting states were used for training with 5-fold cross-validation to optimise the model hyperparameters and yet another 10,000 for testing. We used a regularised ridge regression algorithm to train the linear readout. Testing is done on the best case resulting from the cross-validation. All reported error rates are related to the test data. With 10,000 bits for testing, error rates are reported at a confidence level of about 90%^27^.

### Data availability

The datasets generated and analysed for this work are available from the corresponding author on reasonable request.
